# Prevalence of Soil–Transmitted Helminth Infections and Associated Risk Factors among Schoolchildren in Nakhon Si Thammarat, Thailand

**DOI:** 10.18502/ijpa.v15i3.4210

**Published:** 2020

**Authors:** Pokkamol LAORAKSAWONG, Aubonrat SUNTARALUK, Watcharapong KONGNIL, Pongphan PONGPANITANONT, Penchom JANWAN

**Affiliations:** 1. School of Health Science, Sukhothai Thammathirat Open University, Pakkret, Nonthaburi 11120, Thailand; 2. Department of Medical Technology, School of Allied Health Sciences, Walailak University, Nakhon Si Thammarat 80161, Thailand

**Keywords:** Soil, transmitted helminth infections, *Strongyloides stercoralis*, Hookworm, *Trichuris trichiura*, Schoolchildren

## Abstract

**Background:**

Soil–transmitted helminth infections constitute a public health problem in the rural areas of tropical and subtropical regions, including Thailand. We aimed to determine the prevalence of soil–transmitted helminth infections and underlying risk factors among the schoolchildren living in the rural areas of southern Thailand.

**Methods:**

A cross–sectional survey was conducted between Sep and Nov 2018 in the district of Thasala, Nakhon Si Thammarat, Thailand. A total of 192 children, aged 6–12 yr were enrolled. Each child provided a single stool sample that was subjected to a suite of microscopic diagnoses for soil–transmitted helminth. A questionnaire was administered to determine risk factors of the infections. Logistic regression models were applied to investigate associations.

**Results:**

The overall prevalence of soil–transmitted helminth infections was 3.13%; *Strongyloides stercoralis* 2.08%; hookworm 1.04% and *Trichuris trichiura* 0.52%. Children who cutting fingernails short can prevent soil–transmitted helminth infections highly up to 90% (crude OR = 0.1; 95% confidence interval = 0 – 0.8; *P* = 0.020).

**Conclusion:**

The finding of the study shows a sharp decrease in the prevalence of soil–transmitted helminth among schoolchildren in the southern Thailand in the past two decades with prevalence dropping below 5% for soil–transmitted helminth. However, the prevalence of *S. stercoralis* remained stable over time. These results suggest that the culture method should be used to access strongyloidiasis situation in the older age group who greater contact with soil for agriculturists.

## Introduction

Soil–transmitted helminth (STH) infections are a worldwide disease, especially in the rural areas of tropical and subtropical regions. The infections are mainly caused by five different species of parasitic worms; the roundworm (*Ascaris lumbricoides*), whipworm (*Trichuris trichiura*), hook-worms (*Necator americanus* and *Ancylostoma duodenale*) and threadworm (*Strongyloides stercoralis*). People of all ages are affected by the cycle of these prevalent parasitic infections; however, children are the most affected. The WHO has announced that schoolchildren who live in endemic areas are particularly the group at highest risk of infection and most vulnerable to the pathological consequences (1). Infected children are led to impaired growth and physical development, intellectual and cognitive performance, and maybe die in cases of heavy infection. Nakhon Si Thammarat, the province in the southern part of Thailand is known to be endemic for STH infections especially hookworm infection (2–8). Previous studies showed that hookworm infection among children in rural communities of Nakhon Si Thammarat ranged from 18.0 – 80.0% over the last 2–3 decades (2, 9).

We aimed to investigate the situation of STH infections and to explore the associated risk factors such as environmental factors, sanitation and hygienic practices which favor these infections among schoolchildren in rural area of Nakhon Si Thammarat. The study finding will provide a baseline of information which can be used in the control strategies.

## Materials and Methods

### Study design, study population, sample size, and sampling

A cross–sectional study was conducted on participants aged 6–12 yr in Thaiburi sub– district, Thasala district, Nakhon Si Thammarat province, southern Thailand, from Sep to Nov 2018. Thaiburi sub–district is predominately rural and most residents live in villages as agriculturists growing rice and rubber. The area has covered an area of 28.72 km^2^, an estimated population of 7,554, and the estimated total students between 6–12 yr of age was 351 in 2018. In this study, the sample size was calculated using the single population proportion formula in a finite population condition (10). It was calculated using a prevalence rate (p) of 18.4%, as detailed in a previous study (6), with a 95% confidence interval (z = 1.96) and a 5% margin of error (d = 0.05). The calculated sample size was 140 people. We assumed that the final sample size would end up being reduced by around 25% due to subjects being unable to pass stool on the study date. Thus, we aimed for a sample size of 175. The aged 6–12 yr of participants were selected by a simple random sampling method. A total of 192 subjects were enrolled in this study.

### Ethical approval

The study was approved by the Ethics Committee on Human Rights Related to Research Involving Human Subjects, Walailak University (WUEC–18–057–01). The study’s purpose and procedures were explained to the participants prior to enrolment. Written informed consent was obtained from all participants and participants’ parents or legal guardians before data collection and sampling. All study participants infected with intestinal parasites were referred to the nearby sub–district health–promoting hospital for treatment.

### Sample collection and examination

After, the participants, authorization from parents or legal guardians, sent informed consent to researchers. The participants were asked for stool collection, and on the following day, the plastic containers with at least 10 g of stool were returned to the field staff and transported immediately to the Medical Parasitology Laboratory of School of Allied Health Sciences, Walailak University. The stool samples were examined by; simple direct smear, formalin ethyl acetate concentration technique (FECT) (11), and agar plate culture (APC) (12). Those samples positive for intestinal helminths by at least one laboratory method was considered as positive. Moreover, the parents of participants were interviewed by the questionnaire, which consisted of sociodemographic characteristics and health behavior relating to intestinal helminths infections. The data was entered by two researchers for double–checking the quality of the data.

### Data analysis

The data was processed and analyzed using the SPSS (Chicago, IL, USA) version 16.0 computer software program. The qualitative data were presented in the form of frequencies and percentages while the quantitative data were represented by means and standard deviations. Pearson’s chi–squared test or Fisher’s exact test was used to investigating factors that affect intestinal helminths infection. Logistic regression analysis was used to determine the odds ratio (OR) and its 95% confidence interval (CI) for each associated risk factor. In all cases *P*–value less than 0.05 was taken as statistically significant.

## Results

### The study participants and the prevalence of STH infections

A total of 192 fecal samples were collected from schoolchildren, 52.6% girls and 47.4% boys. The mean age (±SD) of participants was 9.4 (±1.8; range = 6–12 yr), and most of the participants were 6–9 yr old (52.6%) (Table 1). The overall prevalence of intestinal helminthic infections 3.1% (95%CI: 1.1–6.7) including *S. stercoralis* 2.1%, hookworm 1.0%, and *T. trichiura* 0.5%). While most positive cases were single infections, only one child had double infections; *S. stercoralis* and hookworm infections. Both the 6–9 and 10–12 yr old of participants had the prevalence of intestinal helminths infections that were 1.6%.

**Table 1:** Sociodemographic characteristics of participants and analysis factors associated with STH infections

***Variables***	***Total No. (%)***	***Positive No. (%)***	***Crude PR (95% CI)***	***P–value***
Gender				
Male	91 (47.4)	4 (2.1)	1	0.334
Female	101 (52.6)	2 (1.0)	0.4 (0.1 – 2.4)	
Age (yr)				
6 – 9	101 (52.6)	3 (1.6)	1	0.897
10 – 12	91 (47.4)	3 (1.6)	1.1 (0.2 – 5.7)	
Mean ± SD (Min: Max)		9.4 ± 1.8 (6:12)		
Washing hands before meals				
No	85 (44.3)	4 (2.1)	1	0.261
Yes	107 (55.7)	2 (1.0)	0.4 (0.1 – 2.2)	
Eating washed vegetables				
No	13 (6.8)	0 (0)		
Yes	179 (93.2)	6 (3.1)	omitted	
Eating cooked food				
No	35 (18.2)	3 (1.6)		
Yes	157 (81.8)	3 (1.6)	0.2 (0 – 1.1)	0.072
Drinking water treatment				
No	43 (22.4)	1 (0.5)	1	0.723
Yes	149 (77.6)	5 (2.6)	1.4 (0.2 – 12.8)	
Bare foot outside				
No	168 (87.5)	4 (2.1)	1	0.177
Yes	24 (12.5)	2 (1.0)	3.7 (0.6 – 21.6)	
Defecation the feces in toilet				
No	15 (7.8)	1 (0.5)	1	0.468
Yes	177 (92.2)	5 (2.6)	0.4 (0 – 3.7)	
Cutting and looking after the fingernail				
No	43 (22.4)	4 (2.1)		
Yes	149 (77.6)	2 (1.0)	0.1 (0 – 0.8)	0.020
Finger sucking				
No	181 (94.3)	6 (3.1)		
Yes	11 (5.7)	0 (0)	Omitted	
Contact dogs/cats				
No	155 (80.7)	5 (2.6)	1	0.867
Yes	37 (19.3)	1 (0.5)	0.8 (0.1 – 7.4)	
Self–learning about parasitic infection				
No	186 (96.9)	6 (3.1)		
Yes	6 (3.1)	0 (0)	Omitted	
Stool examination				
No	181 (94.3)	5 (2.6)	1	0.331
Yes	11 (5.7)	1 (0.5)	3.5 (0.4 – 33.1)	
Anthelmintic drugs use				
No	178 (92.7)	5 (2.6)	1	0.434
Yes	14 (7.3)	1 (0.5)	2.7 (0.3 – 24.5)	

### Associations of independent variables with STH infections

The distribution of STH infections among study variables was shown in Table 1. According to univariate analysis, only cutting fingernails short was significantly associated with STH infections (Crude OR = 0.1; 95% CI = 0 – 0.8; *P* =0.020). These mean that cutting fingernails short can prevent STH infections was 90%. Regarding the other study variables, more than 77.0% of the participants were done in a good practice, i.e., eating washed vegetables, eating cooked food, drinking water treatment, wearing shoes, defecation the feces in toilet, cutting and looking after the fingernail, no finger sucking, no contact dogs/cats. Only 54.7% of participants washed their hands before eating. In addition, more than 90.0% of the participants had never do self– learning about parasitic infection, annually stool examinations, and used anthelmintic drugs.

## Discussion

The present study showed that the overall prevalence of STH infection was low 3.1% in the Thaiburi sub–district, Thasala district, which dramatically decreased when compare with previous studies over the past two decades that the prevalence among schoolchildren in Nakhon Si Thammarat province, Thailand showed 24.1 – 92.9% (2, 3, 9, 13). Moreover, this prevalence was lower than recent studies, a national survey in the south of Thailand that showed 19.8% (5) and a survey conducted in another district that showed 11.0% (8) of STH infection.

The low prevalence of STH infection in this study may be due to improvements in general living conditions and access to healthcare services, climatic conditions, the local health management, services from government, and the environments. In addition, this may indicate the success of the parasite control project in Thailand by mass treatment, improving the sanitation and personal hygiene of the people in the endemic area.

The present study showed that *S. stercoralis* was the most prevalent (2.1%), followed by hookworm (1.0%), *T. trichiura* being the least prevalent (0.5%) and without *A. lumbricoides* which was not found almost the past two decades. On the contrary, other studies reported hookworm was the most predominant parasite in the Nakhon Si Thammarat province, Thailand (3, 7, 8, 9, 13). This variation may be due to differences in the sampling population, sample size, socioeconomic status of the participants and environmental sanitation, soil type, rainfall, and especially with the parasitological technique used. It was noticed that *S. stercoralis* illustrated the high prevalence with 2.1% by APC technique comparing to the result from the stool specimens detecting by FECT was only 1.0%. This study used a high sensitivity method, APC for *S. stercoralis* detection, and the result can suggest that we should use this method to access strongyloidiasis situation in the older age group who greater contact with soil for agriculturists.

According to previous studies regarding risk factors for intestinal parasitic infection, inadequate personal hygiene increased the risk of infection among primary schoolchildren (8, 14, 15). In this study, children who cut fingernails short had lower infection rates than those who did not. The possible reason might be due to the route of infection through the ground when they play with soil. These studies highlight the need to teach children to get into the recommended habit of cutting fingernails short, under the supervision of teachers and parents to prevent transmission of STH. Moreover, in the overall result, some personal hygiene practices of children were poor. For instance, this study found more than 90% no self–learning about parasitic infection, no stool examination, and no anthelmintic drug use which will be a weak point of parasitic control procedure and should be improved.

## Conclusion

The prevalence of STH infections among primary schoolchildren decrease significantly over for 35 yr in Nakhon Si Thammarat province, Thailand, but they were still prevalent. Therefore, specific control measures are required to interrupt the transmission cycle of STHs. In addition, this is the first study that ascertains out the actual *S. stercoralis* infection rate with the culture method, the best method to detect the prevalence of *S. stercoralis* infection in the past 19 yr.

## Ethical considerations

Ethical issues (Including plagiarism, informed consent, misconduct, data fabrication and/or falsification, double publication and/or submission, redundancy, etc.) have been completely observed by the authors.
